# Microscopic attributes of *Olea europaea* L. leaves and bioclimatology: the case of Galicia, western Iberian Peninsula

**DOI:** 10.3389/fpls.2026.1783046

**Published:** 2026-04-22

**Authors:** Rafael Álvarez, Enrique Escarda-Castro, Miguel Munárriz, Ramón Álvarez-Esteban, Aitor Álvarez-Santacoloma, Sara del Río

**Affiliations:** 1 Molecular Biology Department (Cellular Biology Area), Faculty of Biological and Environmental Sciences, University of León, León, Spain; 2 Economics and Statistics (Statistics and Operational Research), Faculty of Economics and Business, University of León, León, Spain; 3 Biodiversity and Environmental Management Department (Botany Area), Faculty of Biological and Environmental Sciences, University of León, León, Spain; 4 Mountain Livestock Institute (CSIC-ULE), León, Spain

**Keywords:** *Olea europaea*, leaf histology, trichomes, Galician varieties, bioclimatology, varietal differentiation

## Abstract

**Introduction:**

The olive tree (*Olea europaea* L.) is widely recognized for its capacity to persist under conditions of limited water availability, a feature often associated with leaf anatomical and histological traits.

**Methods:**

In the present study, microscopic differences were comparatively analyzed in fully expanded leaves of five olive varieties sampled in Galicia (Brétema, Carapucho, Cobrancosa, Hedreira, and Mansa Gallega) and cultivated under controlled greenhouse conditions. Using light microscopy and scanning electron microscopy, we quantified adaxial cuticle thickness, palisade chlorophyll parenchyma thickness, leaf blade thickness, stomatal density, trichome density, and trichome morphometric traits. Additional qualitative traits were also assessed, including the presence of raphides and trichosclereids, the size of intercellular spaces in the aerenchymatous parenchyma, starch occurrence, the degree of curvature of the distal leaf portion, and the thickness of the vascular bundle sheath in the midrib.

**Results:**

Significant intervarietal differences were detected, allowing the identification of two distinct clusters. Group I (Brétema and Carapucho) was characterized by greater cuticle and leaf blade thickness, higher trichome density, a thinner vascular bundle sheath, and smaller intercellular spaces. Group II (Cobrancosa, Hedreira, and Mansa Gallega) showed lower cuticle and leaf blade thickness, lower trichome density, a thicker vascular bundle sheath, and larger intercellular spaces.

**Discussion:**

These results indicate contrasting histological configurations that may reflect different patterns of environmental adjustment among varieties. The observed traits suggest that Group I displays a more xeromorphic anatomical organization, whereas Group II shows features consistent with comparatively less structurally buffered leaf environments.

**Conclusions:**

Based on these findings and following the global bioclimatic classification system of Rivas-Martínez, the most suitable bioclimatic areas in northwestern Iberia for cultivation of the studied olive varieties are proposed. This study highlights the histological variability of olive leaves and provides useful information for varietal selection in relation to different bioclimatic contexts, including future climate change scenarios.

## Introduction

Bioclimatology studies the relationships between climate and the distribution of living organisms and their communities on Earth. This discipline progressively developed through the association of climatic mean values—especially temperature and precipitation—with the distribution of plants and vegetation types (Rivas-Martínez et al., 2011). Increasingly detailed knowledge of vegetation distribution and compositional change has enabled more precise identification of bioclimatic and vegetation boundaries and the statistical estimation of the climatic thresholds that define them (Rivas-Martínez et al., 2017).

Because it links vegetation patterns to climatic variables, bioclimatology has strong predictive value and has become a useful tool in biodiversity conservation, habitat management, agricultural and forestry planning, food security, and climate change research (Rivas-Martínez et al., 2017). Numerous studies have demonstrated the usefulness of this approach for assessing the potential impacts of climate change on biodiversity, ecosystems, and agroforestry systems. In this context, several investigations on *Vitis vinifera* L. and *Olea europaea* L. have successfully applied this framework (Andrade et al., 2021; Cano et al., 2003; del Río et al., 2021, 2024; Ighbareyeh et al., 2019; Piñar-Fuentes et al., 2024).

Within this broader framework, the olive tree (*Olea europaea* L.) represents one of the most characteristic perennial crops of the Mediterranean Basin. References to olive cultivation can already be found in the writings of Pliny the Elder (77–79 AD). Over time, olive growing has expanded beyond its core Mediterranean range into more humid regions, such as Galicia in the northwestern Iberian Peninsula. In this region, renewed interest in olive cultivation has stimulated efforts to identify and characterize local and relict varieties (Gago et al., 2019, 2024).

The study of Galician olive varieties is relevant not only from an agronomic perspective but also from a genetic and biogeographical standpoint. Previous studies suggest that Galicia may constitute an important reservoir of varietal diversity within *Olea europaea* (Gago et al., 2019). Furthermore, with the exception of Cobrancosa, all varieties analyzed in the present work correspond to relic autochthonous Galician material (Gago et al., 2024). Their anatomical characterization is therefore valuable both for botanical knowledge and for future varietal selection under changing environmental conditions.

Leaves play a central role in photosynthesis, transpiration, and thermal regulation, and their structure may reflect phenotypic variability associated with environmental conditions. Traits such as cuticle thickness, stomatal distribution, trichome density, and the organization of chlorophyll parenchyma can provide useful information on leaf functional structure and plant-environment interactions (Bacelar et al., 2004; Peguero-Pina et al., 2016). At a broader scale, leaf thickness, dry mass per area, and related structural traits are strongly associated with climatic conditions and plant ecological strategies (Niinemets, 2001; Wright et al., 2004). However, most anatomical studies in olive have focused on varieties cultivated under Mediterranean conditions, whereas detailed investigations addressing varieties grown under Atlantic climatic regimes remain scarce.

In this context, the comparative histological study of the varieties Brétema, Carapucho, Hedreira, and Mansa Gallega—recently characterized from molecular and agronomic perspectives (Gago et al., 2024)—together with the cultivar Cobrancosa provides an opportunity to improve their botanical characterization and to identify microscopic traits potentially relevant for varietal comparison. Foliar epidermal micromorphology has also been recognized as informative for taxonomic assessment within Oleaceae (Batool et al., 2025).

The present study comparatively analyzes, using light microscopy and scanning electron microscopy, the microscopic anatomy of leaves from these five *Olea europaea* varieties. Both quantitative variables (such as leaf blade thickness, trichome number and morphology, and stomatal density) and qualitative anatomical traits are evaluated. In addition, as an exploratory case study, these anatomical differences are considered in relation to the most suitable bioclimatic areas for cultivation of the studied varieties in Galicia and adjacent territories.

## Materials and methods

### Plant material and sampling

Fully expanded leaves from five varieties of *Olea europaea* L. were histologically processed. Sampling was carried out on living plants from the collection maintained by the Misión Biológica de Galicia (CSIC) in Pontevedra, Galicia (Spain). All samples were obtained from potted plants grown under greenhouse conditions. All varieties were maintained under identical cultivation conditions, including substrate, irrigation regime, light exposure, and temperature. At the time of sampling, the plants had been grown under these conditions for at least two years.

The varieties studied were Brétema (BR), Carapucho (CA), Cobrancosa (CO), Hedreira (HE), and Mansa Gallega (MA), all of which have been botanically and molecularly characterized (Martins-Lopes et al., 2009; Gago et al., 2024).

One fully expanded leaf was collected from each of six different individuals per variety (n = 6 individuals per variety). Leaves were sampled from the middle portion of the plant, approximately equidistant from the apex and the substrate surface, and care was taken to select leaves with comparable orientation whenever possible.

Samples were fixed in FAA (formaldehyde–acetic acid–alcohol) and subsequently preserved in 70% ethanol. The material was then processed for observation by light microscopy and scanning electron microscopy following the protocols described by Álvarez (2024).

### Light microscopy

Transverse sections were obtained with a scalpel from the median region of the leaves, approximately 5 mm in length per leaf. The fragments were embedded in paraffin following a standard procedure: dehydration through a graded ethanol series, transfer to isoamyl acetate as an intermediate fluid, infiltration with paraffin in an oven at 60 °C, and block preparation using Leuckart bars, paying particular attention to sample orientation. From each block, serial sections 12 µm thick were cut using a paraffin microtome.

Sections from each sample were mounted on six microscope slides (4–5 sections per slide) previously coated with Mayer’s albumin. Slides 1 to 5 (slide 6 was reserved) were deparaffinized in xylene and rehydrated through a descending ethanol series. Slides 1, 3, and 5 were stained with safranin–fast green; slide 2 was stained with Lugol’s solution to detect the presence of amyloplasts; and slide 4 was mounted unstained for epifluorescence observation. All slides were permanently mounted with Entellan.

Observations were carried out using a Nikon E600 microscope, alternately operated in bright-field, polarized light, and epifluorescence modes.

### Scanning electron microscopy

Several fragments (approximately 3 × 3 mm) were obtained from the median region of the leaf blade, both to the right and left of the midrib. Some fragments were used to study the adaxial surface and others the abaxial surface of the leaf blade. Samples were dehydrated through a graded ethanol series. Absolute ethanol was replaced, under appropriate pressure conditions, with liquid CO_2_. Critical point drying was performed, and the samples were coated with gold. Observations were carried out using a JEOL JSM-6480LV scanning electron microscope.

### Measurements and counts

**Figure 1:**
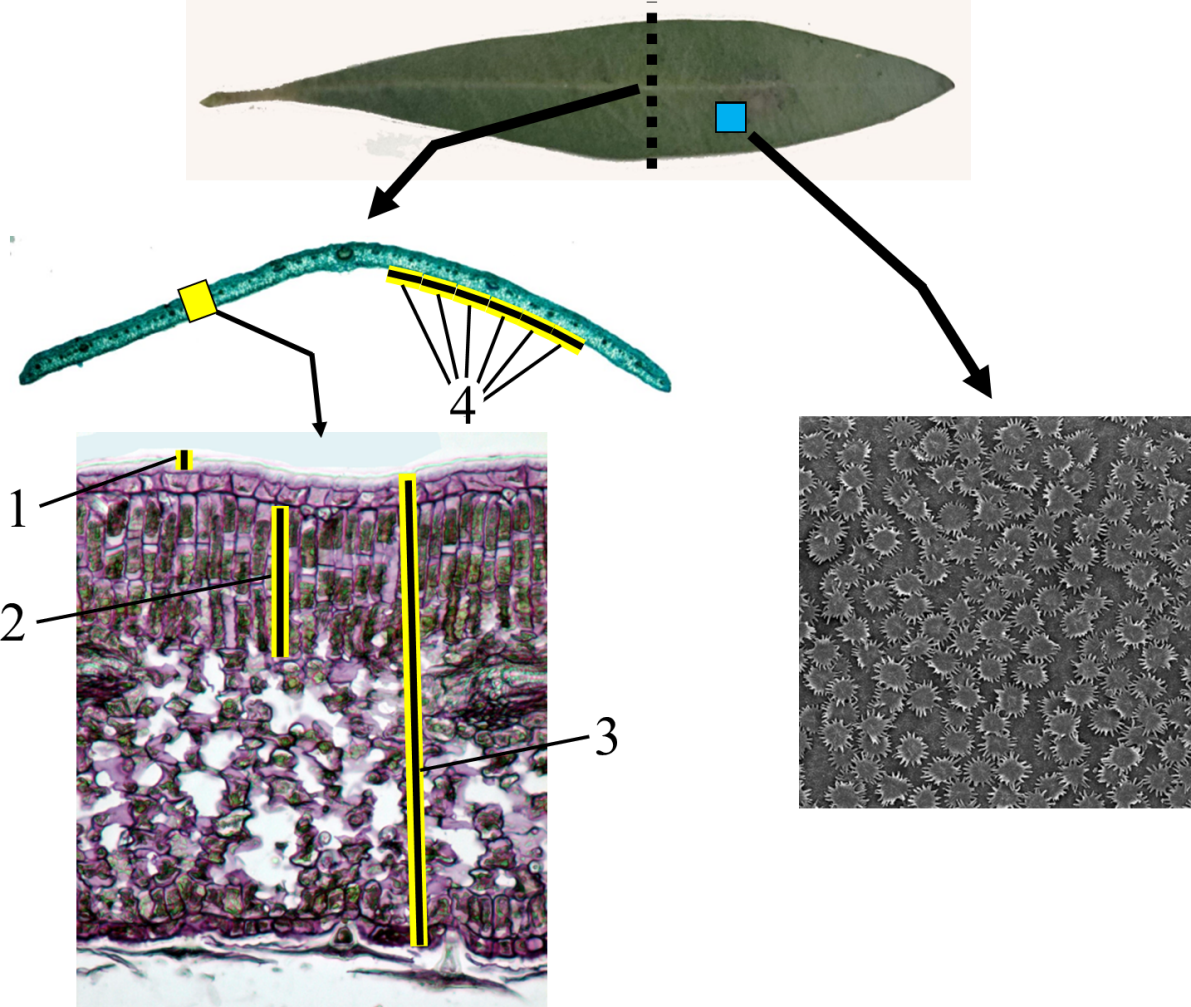
Measurements and counts performed on *Olea europaea* leaves. Bright-field light microscopy: in transverse sections of the median part of olive leaves (dotted line), specifically in the leaf blade (yellow rectangle), the thickness of the following tissues was measured: adaxial cuticle (1), palisade chlorophyll parenchyma (2), and total leaf blade (3). Stomatal density was quantified in six areas of the abaxial epidermis to the right of the midrib (4) and six additional areas to the left of the midrib. Scanning electron microscopy (SEM): in small fragments taken from the median portion of the leaf blade (blue square), trichome density was recorded and trichome attributes were analyzed (see **Figure 2**).

**Figure 2:**
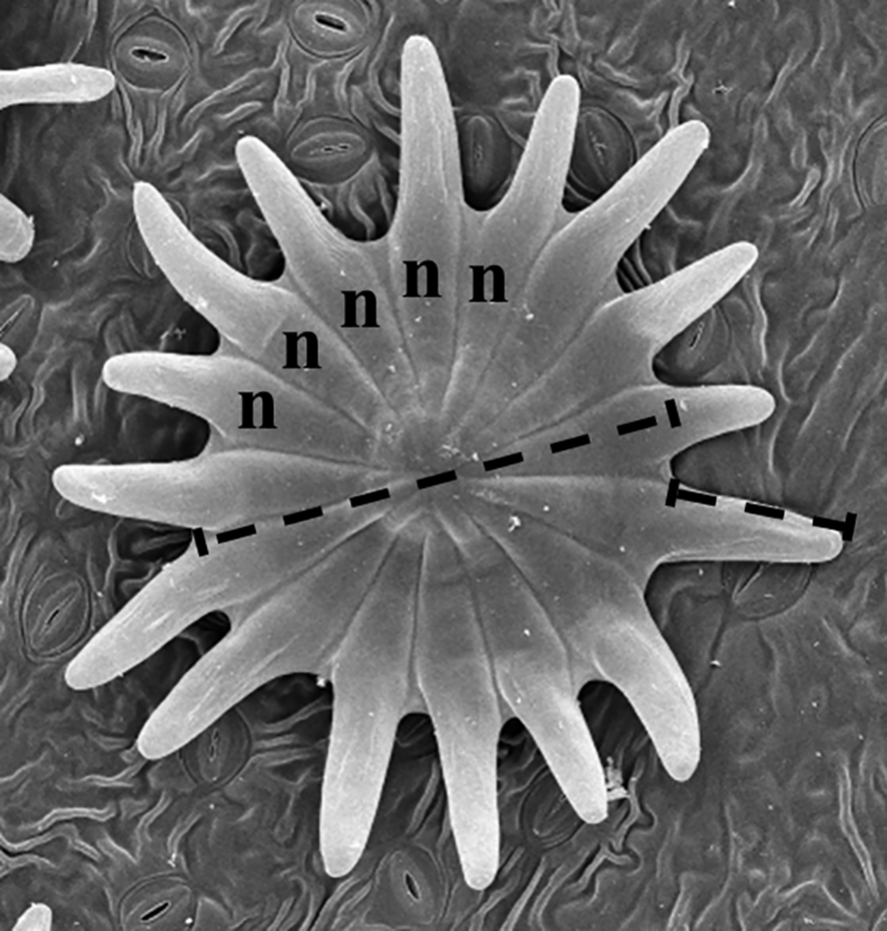
Counts and measurements performed on *Olea europaea* trichomes from both adaxial and abaxial epidermises. The number of cells (n) forming the trichome scale or shield is indicated (only five cells are labelled for clarity). The long line represents the diameter of the central disc of the scale, i.e., the portion where the cells are tightly packed. The short line indicates the length of the scale cells that extend beyond the central disc (here referred to as trichome arms).

Unless otherwise stated, counts and measurements were performed on micrographs. Measurements were obtained using AxioVision software. The variables analyzed were as follows (**Figure 1**):

- Cuticle thickness (µm): Measurements were taken from three leaves per variety, with 6–9 measurements per leaf on both sides of the midrib (total of 24 measurements per variety).
- Thickness of palisade chlorophyll parenchyma (µm): Same sampling design as above, resulting in 24 measurements per variety.
- Leaf blade thickness (µm): Same sampling design as above, resulting in 24 measurements per variety.
- Number of stomata: Stomatal counts were performed on microscopic preparations using a bright-field optical microscope equipped with an ocular reticle. On both sides of the midrib, six consecutive stomatal counts were carried out on the abaxial epidermis, starting 250 µm away from the midrib. Each count covered a 250 µm-long area. In total, 6 counts × 250 µm = 1500 µm were analyzed on one side of the midrib and another 1500 µm on the opposite side, resulting in stomatal counts over 3000 µm of epidermis. Twelve counts were performed per leaf, with three leaves per variety, resulting in 36 counts per variety.
- Number of trichomes: Trichome density on the adaxial and abaxial surfaces was determined over an area of 0.2 mm^2^ using scanning electron microscopy (250×). Six counts were performed on a single leaf (three on each side of the midrib). Trichome density was obtained by dividing the number of trichomes by the analyzed surface area.
- Trichome characteristics: In five adaxial and five abaxial trichomes (five trichomes per variety), the following features were analyzed (**Figure 2**):
  - number of cells forming the shield or scale,
  - diameter of the central disc (µm) (five measurements per trichome),
  - length of the arms (µm) (five randomly selected arms),
  - total trichome diameter, mathematically calculated as the mean value of the central disc diameter plus the mean arm length.
- Subjective variables: Semi-quantitative assessment (high, intermediate, low, absent) of:
  - presence of raphides,
  - presence of trichosclereids,
  - size of intercellular spaces (meatuses) in the aerenchymatous parenchyma,
  - presence of starch,
  - increase in trichosclereids in the distal portion compared to the leaf blade,
  - degree of curvature of the distal portion,
  - number of layers in the vascular bundle sheath.

### Statistical analysis

Using data from leaf measurements (cuticle thickness, palisade chlorophyll parenchyma thickness, leaf blade thickness), stomatal density, trichome density, and trichome attributes, the null hypothesis of normality was rejected for nine of the fourteen variables based on the Lilliefors and Shapiro–Wilk tests. Consequently, a non-parametric Kruskal–Wallis test was applied to the five independent samples, followed by a *post hoc* Dunn (1964) test with Holm (1979) correction, using the *rstatix* R package (Kassambara, 2023), to identify homogeneous groups of varieties. The *cld* package (Gegzna, 2025) was used to generate compact letter displays for pairwise *post hoc* comparisons.

Mean values for each variable and variety of *O. europaea* were calculated from the analyzed data. As the variables were expressed in different units, they were scaled prior to computing the Euclidean distance matrix. Hierarchical cluster analysis was then performed using Ward’s aggregation method. Continuous quantitative variables were described using means and standard deviations, while qualitative variables were described using proportions and their corresponding 95% confidence intervals. In all analyses, a significance level of α = 0.05 was applied. The Wilcoxon test for two independent samples was performed using the *wilcox_test* function from the *rstatix* package to compare the two groups obtained from the cluster analysis. All statistical analyses were conducted using R software v.4.5.2.

### Bioclimatic analysis

Bioclimatic maps previously generated in other studies were used to identify areas suitable for the cultivation of the studied varieties. These maps incorporate information on macrobioclimates, bioclimates, bioclimatic variants, bioclimatic belts (thermotypes and ombrotypes), and continentality, according to the classification system of Rivas-Martínez et al. (2011, 2017).

## Results

### General leaf histology

**Figure 3:**
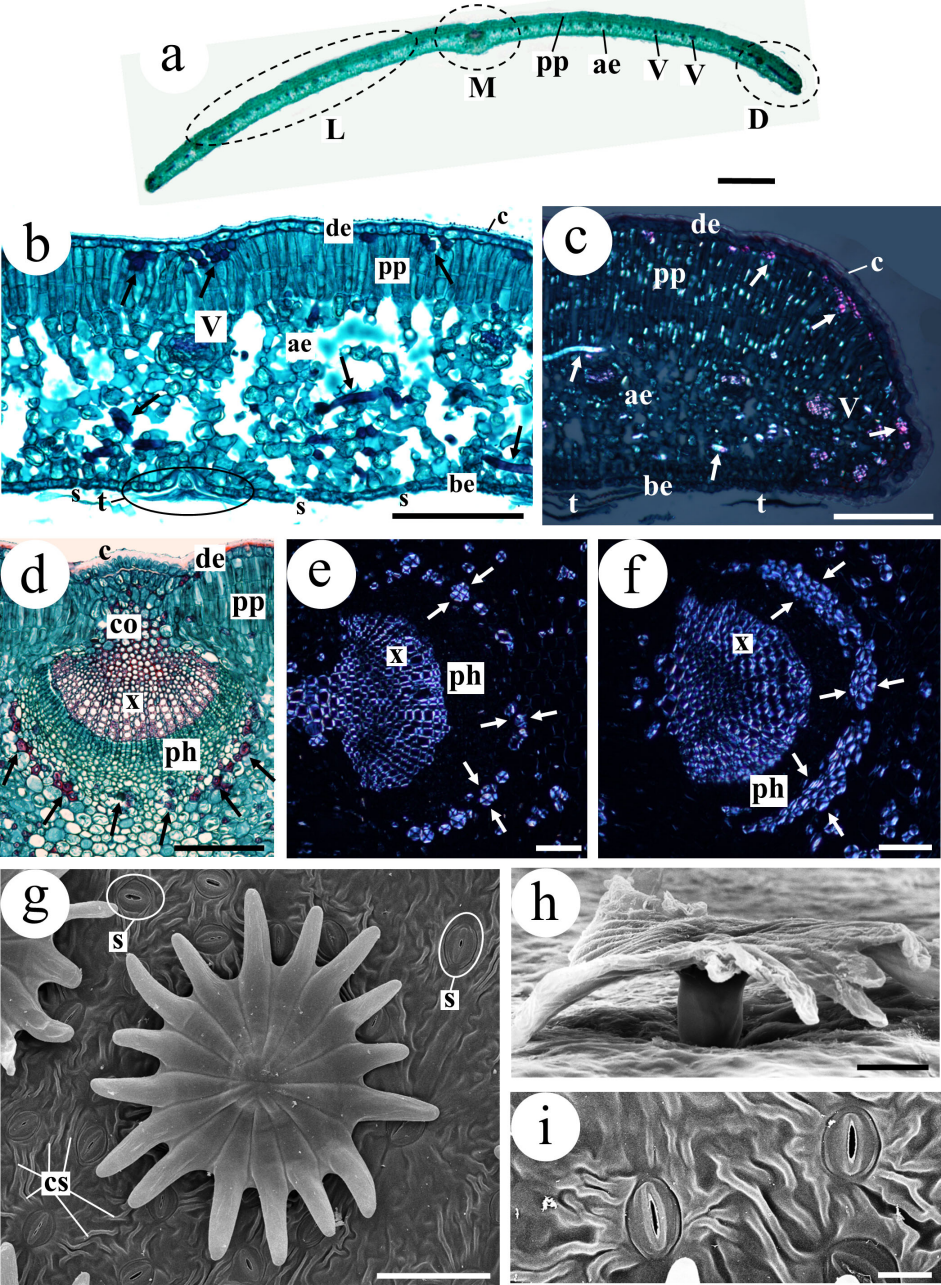
Leaf anatomy of *Olea europaea*. **(a)** Transverse section of a leaf showing a prominent midrib (M) and second-order veins (V) within the leaf blade (L). Palisade chlorophyll parenchyma (pp) is located adaxially, whereas aerenchyma (ae) is located abaxially. **(b)** Leaf blade showing stomata (s) exclusively in the abaxial epidermis (be), together with characteristic olive trichomes (t). The adaxial epidermis (de) presents a conspicuous cuticle. Large intercellular spaces (meatuses) are visible in the aerenchyma. Arrows indicate fragments of trichosclereids. **(c)** Distal portion of the leaf. Bright (anisotropic) spots in parenchyma cells correspond to calcium oxalate inclusions (raphides). Arrows indicate fragments of trichosclereids. **(d–f)** Midrib. **(d)** Arrows indicate transverse sections of fibres forming the fascicular sheath. **(e)** Arrows indicate a thin fascicular sheath. **(f)** Arrows indicate a thick fascicular sheath. **(g, h)** Multicellular peltate scaly trichome characteristic of olive leaves. **(g)** Surface view of the trichome; stomata (s) and epicuticular striations (cs) are visible in the epidermis. **(h)** Lateral view of one trichome showing the stalk. **(i)** Detail of two stomata and epicuticular striae. **(a, c–e, h)** Brétema variety; **(b, f, g, i)** Hedreira variety. Staining: **(a, b, d)** safranin–fast green. Microscopy: **(a, b, d)** bright-field light microscopy; **(c, e, f)** polarized light microscopy; **(g–i)** SEM. ae, aerenchyma; be, abaxial epidermis; c, cuticle; co, collenchyma; cs, cuticular striations; D, distal portion of the leaf; de, adaxial epidermis; L, leaf blade; M, midrib; ph, phloem; pp, palisade chlorophyll parenchyma; s, stoma; t, trichome; V, vascular bundle; x, xylem. Scale bars: **(a)** 1 mm; **(b–g)** 50 µm; **(h)** 20 µm; **(i)** 10 µm.

Leaves of *Olea europaea* show the typical histological organization of dicotyledonous leaves (**Figure 3a**). Macroscopically, the leaf presents a prominent midrib from which second-order veins extend throughout the blade. The leaf blade is dorsiventral, with palisade chlorophyll parenchyma on the adaxial side and aerenchymatous parenchyma on the abaxial side (**Figures 3a, b**). Leaves are hypostomatic, with stomata occurring exclusively on the abaxial surface (**Figure 3b**). Stomata are flush with the epidermal surface and are anomocytic, lacking subsidiary cells associated with the guard cells.

### Quantitative and qualitative differences among varieties

**Figure 4:**
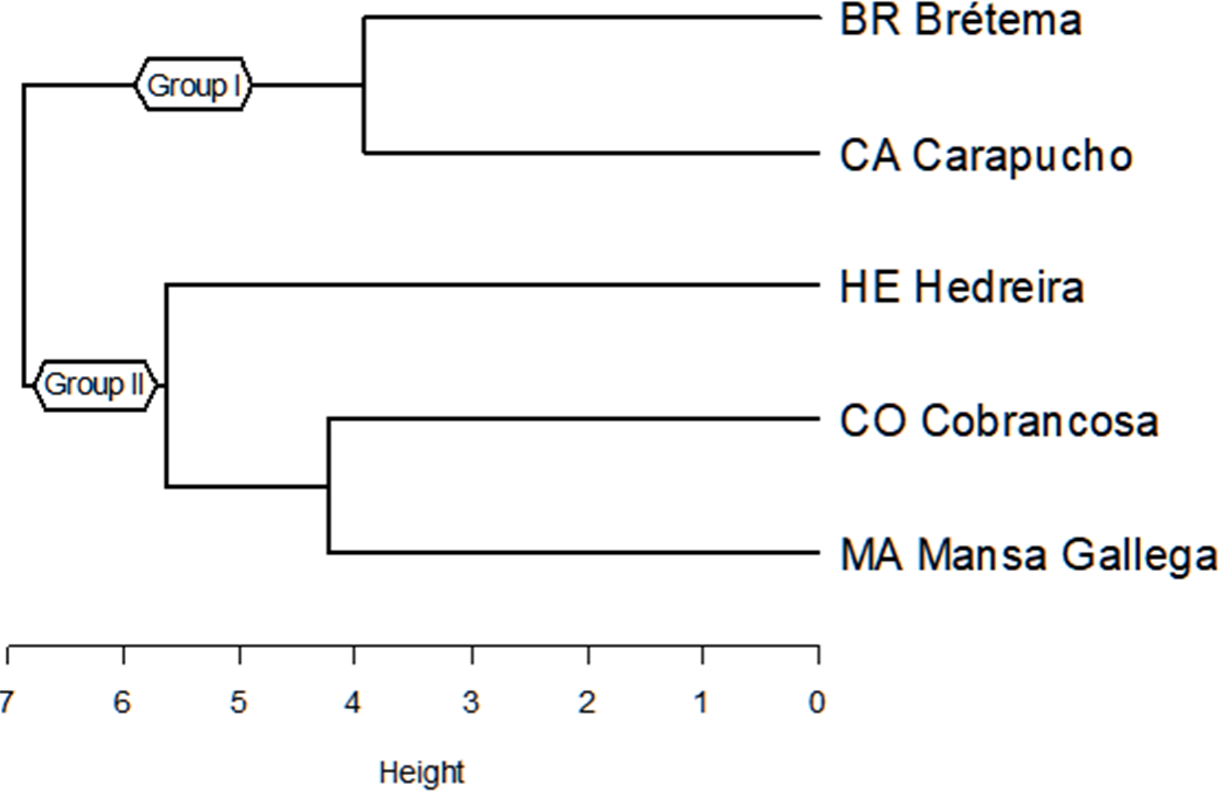
Hierarchical cluster analysis of the five olive varieties based on the analyzed anatomical variables. Two groups were identified: a more homogeneous group consisting of Brétema (BR) and Carapucho (CA) (Group I), and a more heterogeneous group comprising Mansa Gallega (MA), Cobrancosa (CO) and Hedreira (HE) (Group II).

Significant differences were observed for most analyzed variables (**Table 1**). Hierarchical clustering of the complete dataset revealed two distinct groups of varieties: Group I (Brétema and Carapucho) and Group II (Cobrancosa, Mansa Gallega, and Hedreira) (**Figure 4**).

**Table 1:** Mean values and standard deviations (italics, below the main value) of the variables analyzed in the five olive varieties: Brétema (BR), Carapucho (CA), Cobrancosa (CO), Hedreira (HE) and Mansa Gallega (MA).

Variables	BR	CA	CO	HE	MA	Mean
Adaxial cuticle THK (μm)	2.66^a,b^	2.77^a^	2.19^b,c^	1.84^c^	2.50^a,b^	2.38***
	*0.74*	*0.56*	*0.79*	*0.62*	*0.48*	*0.72*
THK palisade parenchyma (μm)	47.15^a^	41.34^a^	23.72^b^	24.14^b^	40.84^a^	35.44***
	*5.27*	*4.14*	*2.30*	*3.83*	*3.69*	*10.45*
Leaf blade THK (μm)	130.97^a^	120.60^a,b^	84.43^c^	97.49^c^	113.44^b^	109.39***
	*6.56*	*7.14*	*8.32*	*8.58*	*5.31*	*18.11*
Stomatal density (no. mm^-1^)	4.48^a,b^	5.52^a^	2.04^c^	3.78^b^	4.00^b^	3.96***
	*3.78*	*3.97*	*2.54*	*3.54*	*3.28*	*3.63*
No. trichomes adaxial (mm^2^)	22.50^a,b^	29.17^a^	8.33^b,c^	12.50^a,b,c^	7.50^c^	16.00***
	*6.89*	*4.92*	*4.08*	*5.24*	*4.18*	*9.86*
No. trichomes abaxial (mm^2^)	72.50^a^	75.00^a^	28.33^b^	56.67^a,b^	40.83^b^	54.67***
	*5.24*	*5.48*	*5.16*	*9.83*	*3.76*	*19.21*
Trichome attributes						
No. cells - adaxial	28.4^a^	18.4^b^	28.40^a^	18.40^b^	21.20^a,b^	22.96***
	*3.51*	*2.88*	*2.07*	*2.88*	*2.59*	*5.32*
No. cells - abaxial	32.00^a^	29.00^a,b^	31.20^a^	21.20^b^	29.60^a,b^	28.60***
	*0.00*	*2.00*	*0.84*	*0.84*	*3.36*	*4.27*
Central disc - adaxial (µm)	125.00^a^	107.00^a,b^	114.00^a,b^	108.00^a,b^	101.00^b^	111.00**
	*10.00*	*12.04*	*6.52*	*2.74*	*12.94*	*12.08*
Central disc - abaxial (µm)	149.00^a^	139.00^a,b^	147.00^a,b^	117.00^b^	156.00^a^	141.60***
	*7.42*	*4.18*	*12.04*	*8.37*	*16.73*	*16.82*
Arms - adaxial (µm)	12.12^b^	17.96^a^	7.92^c^	16.04^a,b^	11.32^b^	13.07***
	*4.31*	*6.12*	*4.25*	*6.60*	*3.73*	*6.18*
Arms - abaxial (µm)	28.56^a,b^	35.08^a^	14.64^c^	29.88^a,b^	23.36^b^	26.30***
	*9.43*	*10.84*	*8.00*	*11.01*	*6.73*	*11.53*
*Disc and arms - adaxial (µm)*	*137.12*	*124.96*	*121.92*	*124.04*	*112.32*	*124.07*
*Disc and arms - abaxial (µm)*	*177.56*	*174.08*	*161.64*	*146.88*	*179.36*	*167.90*
Unquantified variables						
Raphides	+++	+	++	++	++	
Trichosclereids	++	++	+	+++	++	
Intercellular spaces	+	+	++	+++	+	
Starch	–	–	–	–	–	
More trichosclereids - distal portion	–	+	+	+	–	
Distal portion curvature	+	++	+	^+^	++	
Bundle sheath	+	+	++	++	++	

Asterisks indicate the level of statistical significance of the Kruskal–Wallis test among the five varieties (*p < 0.1; **p < 0.05; ***p < 0.01). Different letters indicate statistically significant differences among varieties according to Dunn’s *post hoc* test with Holm correction. The letter “a” corresponds to the group with the highest value of the variable, and “z” to the group with the lowest value. For ordinal variables, “–” indicates absence, while “+”, “++” and “+++” indicate increasing presence or magnitude. Variables (from top to bottom) include: adaxial cuticle thickness, palisade chlorophyll parenchyma thickness, leaf blade thickness, number of stomata, number of trichomes on the adaxial and abaxial epidermis, and trichome attributes of both epidermal surfaces: number of cells forming the scale or shield, central disc diameter, arm length, and total trichome size (disc + arms). Additional qualitative variables are also indicated: presence of raphides, presence of trichosclereids, size of aerenchyma meatuses, presence of amyloplasts, relative abundance of trichosclereids in the distal portion, curvature of the distal portion, and thickness of the fascicular sheath.

**Figure 5:**
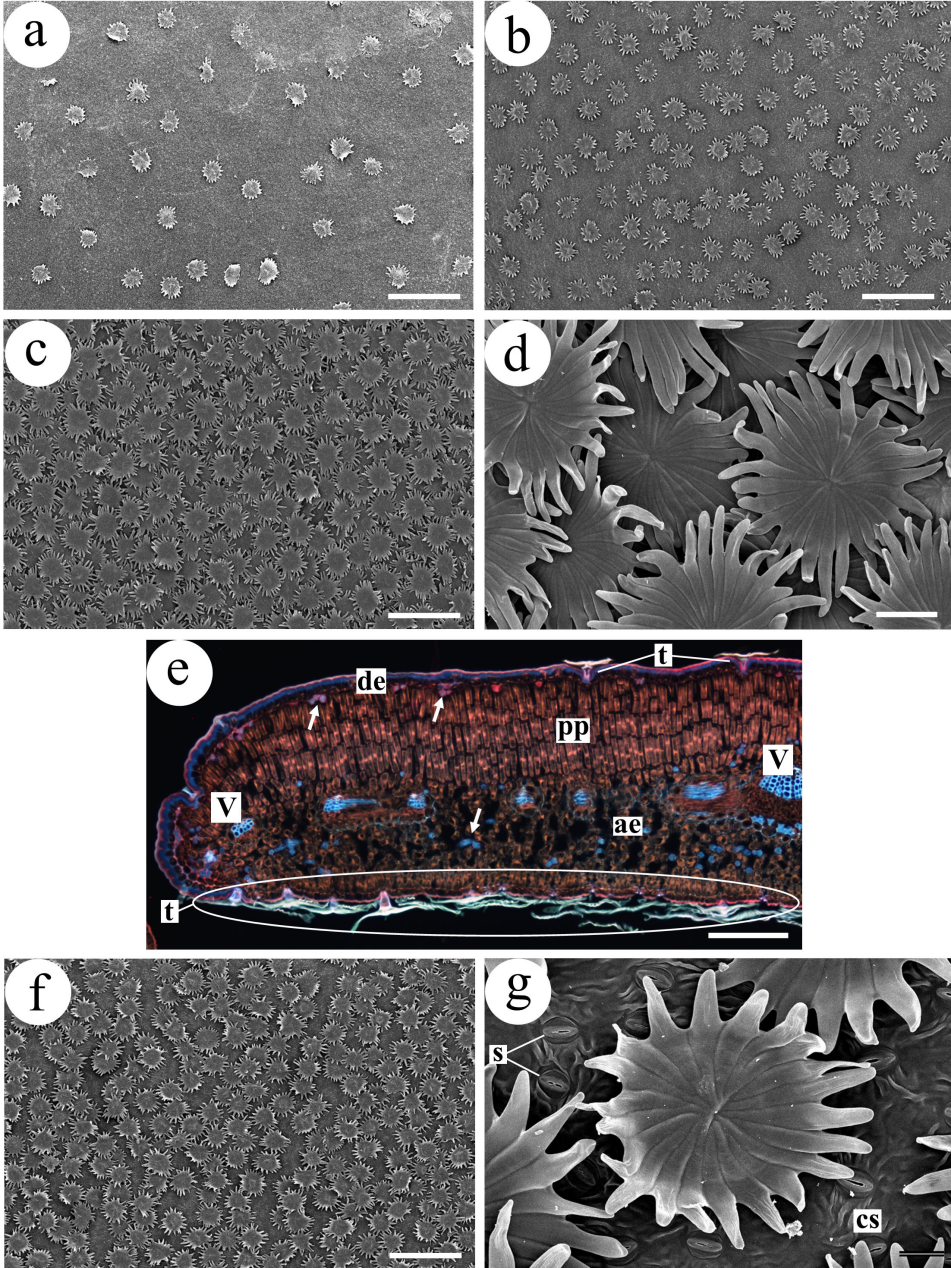
Leaf surface and internal anatomy of *Olea europaea*. **(a,b)** Adaxial leaf surface. In **(a)** very few trichomes are present; in **(b)** trichomes are more abundant, but always fewer than on the abaxial epidermis. **(c,d)** Abaxial surface with abundant trichomes, which cover the entire surface and appear arranged in several layers **(d)**, preventing direct observation of the epidermal surface. **(e)** Leaf blade and distal portion. Numerous trichomes (t) are present on the abaxial epidermis, whereas only two trichomes are visible on the adaxial surface (de). Arrows indicate fragments of trichosclereids. Compare with **Figure 3b** in terms of leaf blade thickness, aerenchyma meatus size and trichome abundance. **(f, g)** Abaxial surface with abundant trichomes; in **(g)** the epidermal surface between trichomes is visible. **(a)** Hedreira; **(b–d)** Carapucho; **(e)** Brétema; **(f, g)** Cobrancosa. **(a–d, f, g)** SEM; **(e)** epifluorescence microscopy. ae, aerenchyma; cs, cuticular striations; de, adaxial epidermis; pp, palisade chlorophyll parenchyma; t, trichome; V, vascular bundle. Scale bars: **(a–c, f)** 300 µm; **(d, e)** 50 µm; **(g)** 20 µm.

The leaf blade shows a mean thickness of 109.39 µm (125.79 µm in Group I and 98.45 µm in Group II) (**Table 2**). From the adaxial to the abaxial surface, the following tissues can be distinguished (**Figures 3a, b**, **5e**):

**Table 2:** Mean values of Group I (Brétema and Carapucho) and Group II (Cobrancosa, Mansa Gallega and Hedreira).

Variables	Group I BR-CA	Group II HE-CO-MA	Mean
Adaxial cuticle THK (μm)	2.72	2.18	2.39***
THK palisade parenchyma (μm)	44.25	29.57	35.44***
Leaf blade THK (μm)	125.79	98.45	109.39***
Stomatal density (no. mm^-1^)	5.00	3.27	3.96***
No. trichomes adaxial (mm^2^)	25.83***	9.44***	16.00***
No. trichomes abaxial (mm^2^)	73.75***	41.94***	54.67***
Trichome attributes			
No. cells - adaxial	23.40***	22.67**	22.96
No. cells - abaxial	30.50***	27.33**	28.60
Central disc - adaxial (µm)	116.00***	107.67***	111.00*
Central disc - abaxial (µm)	144.00***	140.00***	141.60
Arms - adaxial (µm)	15.04***	11.76***	13.07
Arms - abaxial (µm)	31.82***	22.63***	26.30***
*Disc and arms - adaxial (µm)*	*131.04*	*119.43*	*124.07*
*Disc and arms - abaxial (µm)*	*175.82*	*162.63*	*167.90*

Significance in the “Mean” column indicates differences in central tendency between Groups I and II using the Wilcoxon test. Significance in the “Group I” and “Group II” columns indicates differences between adaxial and abaxial values within each group. *p < 0.1; **p < 0.05; ***p < 0.01. Variables include (from top to bottom): adaxial cuticle thickness, palisade chlorophyll parenchyma thickness, leaf blade thickness, number of stomata, number of trichomes on the adaxial and abaxial epidermis, and trichome attributes of both epidermal surfaces: number of cells forming the scale or shield, central disc diameter, arm length, and total trichome size (disc + arms).

- Adaxial epidermis: Uniseriate, composed of more or less isodiametric cells. The cuticle is conspicuous, with a mean thickness of 2.39 µm, being thinner in the varieties Cobrancosa and Hedreira. Overall, the cuticle is significantly thicker in Group I (2.72 µm) than in Group II (2.18 µm). No stomata are present. Few multicellular peltate scaly trichomes occur on this surface (**Figures 5a, b**), with a mean density across varieties of 16.00 trichomes mm^−2^.
- Palisade chlorophyll parenchyma: Mean thickness of 35.44 µm. It is composed of prosenchymatic cells arranged in 1–3 layers, containing abundant chloroplasts, tannins, and occasionally raphides (calcium oxalate crystals) (**Figure 3c**). A significant difference was detected between Group I (44.25 µm) and Group II (29.57 µm), although Mansa Gallega (MA) showed values similar to those of Group I (40.84 µm) (**Table 1**).
- Aerenchymatous parenchyma: Cells contain chloroplasts and tannins and show conspicuous intercellular spaces of variable size, generally larger in Group II, although the MA variety is similar to Group I (**Figures 3b**, **5e**). In some cases, 1–2 layers of palisade chlorophyll parenchyma composed of shorter cells are present adjacent to the abaxial epidermis.
- Abaxial epidermis: Uniseriate, composed of isodiametric cells, with a cuticle bearing epicuticular striations (**Figures 3g, i**, **5g**). Anomocytic stomata are present (**Figures 3b, g, i**, **5g**), with the lowest stomatal density observed in the variety Cobrancosa (CO). Numerous multicellular peltate scaly trichomes occur on this surface (**Figures 3g, h**), with a mean density of 54.67 trichomes mm^−2^. Trichome density is significantly higher in Group I (73.75 trichomes mm^−2^) than in Group II (41.94 trichomes mm^−2^) (**Figures 5c–g**). In Group I, trichomes are arranged in several overlapping layers, preventing visualization of the epidermal surface in surface views (**Figure 3d**). In Group II, trichomes are also significantly more abundant than on the adaxial surface but are arranged in a single layer, allowing the epidermal surface between them to be observed (**Figure 3g**).
- Leaf blade as a whole: Trichosclereids and second-order veins are present throughout the blade (**Figures 3a, b**, **5e**). Trichosclereids occur across the entire leaf but are particularly evident in transverse sections, where they are arranged subepidermally beneath the adaxial epidermis (**Figures 3b**, **5e**). Second-order veins show anatomical features similar to those of the midrib, although with fewer supporting elements (collenchyma and sclerenchyma). Raphides are observed throughout the leaf except in vascular tissues. No amyloplasts were detected.

Histologically, the midrib shows the following features (**Figures 3d–f**):

- Epidermis: Uniseriate on both adaxial and abaxial surfaces, with cells that may be cylindrical with papillae, cubic, or flattened. Stomata are absent. The cuticle is conspicuous, and few trichomes are present.
- Vascular bundle: A closed collateral vascular bundle surrounded by a fibrous vascular bundle sheath (**Figure 3d**). In Group I, the vascular bundle sheath is thin (1–2 cell layers), whereas in Group II it is thicker (3–4 cell layers).
- Ground tissues: Beneath both epidermises, 3–5 layers of annular collenchyma are present, followed inward by 3–5 layers of storage parenchyma up to the vascular bundle. External to the vascular bundle, trichosclereids are generally observed.

The distal portion of the leaf (**Figure 3c**) appears either as a straight continuation of the blade or curved to varying degrees toward the abaxial surface. Compared to the rest of the blade, it exhibits a thicker cuticle, aerenchymatous parenchyma with smaller intercellular spaces, and a higher abundance of trichosclereids.

The trichomes characteristic of *O. europaea* are stalked structures supporting a group of cells that, depending on the author, have been described as forming a shield, umbrella, scale, or star (**Figures 3g, h**, **5d, g**). These cells (approximately 18–32) are radially arranged, forming a central disc without intercellular spaces. Peripheral to this disc, the cells become individualized, constituting what are referred to here as the trichome arms.

Both the diameter of the central disc and the length of the trichome arms are significantly greater on the abaxial surface than on the adaxial surface. Consequently, the total trichome diameter is larger on the abaxial surface (mean 167.90 µm) than on the adaxial surface (mean 124.07 µm) (**Table 1**).

## Discussion

In the present study, fully expanded leaves from five *Olea europaea* varieties were examined microscopically. All varieties displayed the general anatomical features commonly described for olive leaves (Bacelar et al., 2004; Koudounas et al., 2015; Najmaddin, 2016; Hmmam et al., 2018).

At the same time, several anatomical traits differed consistently among varieties. These differences allowed the recognition of two groups: Brétema and Carapucho (Group I) and Cobrancosa, Mansa Gallega, and Hedreira (Group II). Rather than directly demonstrating physiological drought tolerance, these findings provide a comparative anatomical framework that may help interpret structural differences among varieties. Comparable anatomical responses of olive leaves to drought stress have also been described in other cultivars (Bosabalidis and Kofidis, 2002).

The cuticle acts as an important barrier against water loss. A thicker adaxial cuticle may reduce cuticular transpiration, whereas thinner cuticles provide lower resistance to water diffusion (Karabourniotis et al., 2001; Rosado and Holder, 2013). In this study, the thinnest cuticles were observed in Hedreira and Cobrancosa.

Epicuticular striations observed on the abaxial epidermis have been reported in olive and other plants from environmentally demanding habitats (Bacelar et al., 2004; Álvarez et al., 2008). These structures may contribute to reflecting radiation, reducing leaf overheating, and reinforcing the cuticle under water deficit conditions (Fahn, 1986; Karabourniotis et al., 2001; Bacelar et al., 2004).

Trichomes contribute to the development of the leaf boundary layer and may influence leaf–environment interactions by reducing convective exchange and buffering environmental stress (Ehleringer and Mooney, 1978; Roth-Nebelsick et al., 2014; Taiz et al., 2015). This interpretation is also supported by recent evidence showing that leaf trichomes reduce boundary layer conductance (Pierce et al., 2022). The higher trichome density observed in Group I may therefore represent a structural trait potentially associated with enhanced buffering against environmental stresses such as drought, wind exposure, or salinity. However, these interpretations should be considered anatomical inferences rather than direct physiological evidence. Leaf surface structures may also influence foliar water and solute absorption processes (Fernández et al., 2021).

Several additional microscopic features were qualitatively assessed, including the presence of raphides and trichosclereids, the relative size of intercellular spaces in the aerenchymatous parenchyma, starch presence, the degree of curvature of the distal leaf portion, variation in trichosclereid abundance in the distal region, and the thickness of the vascular bundle sheath in the midrib. Considerable heterogeneity among varieties was observed for these non-quantified traits, except for the consistently thick vascular bundle sheath found in Group II varieties (Mansa Gallega, Cobrancosa, and Hedreira).

The presence of trichosclereids in the mesophyll and a sclerenchymatous vascular bundle sheath in the midrib has a clear mechanical function and is characteristic of xeromorphic leaves (Fahn, 1986; Evert and Eichhorn, 2006; Taiz et al., 2015). Additionally, Karabourniotis et al. (1994) demonstrated that olive trichosclereids can transmit visible light into the mesophyll, enhancing photosynthetic efficiency under high irradiance.

Calcium oxalate crystals are a well-known feature in plant tissues. Cells avoid calcium toxicity by precipitating excess calcium as calcium oxalate (Álvarez, 2024). In photosynthetically active cells, excessive calcium may interfere with photosynthesis; therefore, raphide formation represents an effective mechanism for maintaining calcium homeostasis (Azcón-Bieto and Talón, 2008; Tooulakou et al., 2016). Under stress conditions such as drought or stomatal closure, calcium oxalate crystals may be degraded by oxalate oxidase, providing an internal carbon source for photosynthesis when external CO_2_ availability is limited.

Microscopic observation of the degree of curvature of the distal leaf portion among varieties likely reflects differences in overall leaf exposure to environmental conditions. Future studies integrating microscopic observations with macroscopic and anatomical analyses may help elucidate relationships between leaf folding and specific histological configurations. In the present study, no clear relationship was observed between distal curvature and trichosclereid abundance.

The absence of amyloplasts in all five varieties, as revealed by Lugol-stained sections, is consistent with previous descriptions of olive as a sclerophyllous and xeromorphic species. Whereas mesophytes typically accumulate transient starch as an energy reserve, xerophytes maintain low or negligible foliar starch levels, favoring soluble sugars instead (Bustan et al., 2011; Tsamir-Rimon et al., 2020; Li et al., 2025).

Smaller intercellular spaces in the aerenchymatous parenchyma may be associated with a more compact mesophyll organization, whereas larger spaces, as observed in Hedreira and Cobrancosa, may reflect a comparatively looser internal structure (Tognetti et al., 2006).

Scanning electron microscopy allowed detailed analysis of trichome characteristics on both leaf surfaces, including the number of cells forming the scale or shield, the diameter of the central disc, and the length of the individualized peripheral cells (here termed trichome arms; see **Figure 2**). Significant differences were observed between adaxial and abaxial trichomes for all three variables: abaxial trichomes showed a higher number of cells, larger diameters, and longer arms. Overall, abaxial trichomes were larger than adaxial ones. The smallest adaxial trichomes were found in Mansa Gallega, whereas Hedreira showed the smallest abaxial trichomes.

Few studies have addressed trichome size in olive leaves, and reported values vary depending on leaf age, variety, and environmental exposure. The trichome sizes recorded in the present study (ranging from 112.32 μm in adaxial trichomes of Mansa Gallega to 179.36 μm in abaxial trichomes of the same variety) are consistent with the limited available literature. Fernández et al. (2024) reported a mean trichome diameter of 157 μm in the cultivar Arbequina, without distinguishing between leaf surfaces.

No specific data are available regarding the number of cells forming the trichome scale or the length of the trichome arms. Future research should address these traits in relation to varietal differences and environmental adaptation. Given the importance of the boundary layer in tolerance to drought, wind, and salinity, it would be particularly interesting to investigate whether trichome arm length influences boundary layer dynamics. Such studies could benefit from interdisciplinary collaboration with fluid dynamics specialists to analyze how trichome architecture affects airflow, water retention, and mist capture at and above the boundary layer.

Hierarchical clustering of both qualitative and quantitative variables yielded two distinct groups of varieties: a homogeneous Group I comprising Brétema and Carapucho, and a more heterogeneous Group II comprising Cobrancosa, Mansa Gallega, and Hedreira (**Figure 4**).

Group I is characterized by thicker leaves, higher trichome density on both leaf surfaces, and a thinner vascular bundle sheath. It also shows greater adaxial cuticle thickness, thicker palisade chlorophyll parenchyma, and smaller intercellular spaces in the aerenchymatous parenchyma.

Group II shows thinner leaves, lower trichome density on both surfaces, and a thicker vascular bundle sheath, together with thinner cuticles, reduced palisade chlorophyll parenchyma thickness, and larger intercellular spaces.

Significant differences between the two groups were observed (**Table 2**). Group I exhibited thicker adaxial cuticles, palisade chlorophyll parenchyma, and leaf blades, higher stomatal and trichome densities, and longer trichome arms. Additionally, Group I showed smaller intercellular spaces and a thinner vascular bundle sheath.

Overall, Group I shows a comparatively more xeromorphic anatomical organization, which may be consistent with improved structural adjustment to conditions of higher atmospheric demand, including drought, wind exposure, and salinity. The thicker boundary layer potentially associated with higher trichome density may contribute to reducing transpiration and buffering leaf–environment exchange. From a bioclimatic perspective, this group may therefore be better suited to windy and/or saline environments with high oceanicity.

Group II may be better suited to more continental environments, less affected by salinity and wind but still subject to seasonal drought.

This grouping does not fully coincide with the macroscopic leaf classifications proposed by Gago et al. (2024), who related leaf morphology to environmental adaptation. For example, Brétema exhibits broader, elliptic leaves that may enhance thermal dissipation; Carapucho shows good tolerance to thermal variability and coastal winds; Hedreira presents intermediate traits; Mansa Gallega shows leaf traits associated with efficient humidity management; and Cobrancosa is known for resistance to hot and dry conditions.

As suggested by Bacelar et al. (2004), future studies integrating macroscopic and microscopic analyses would improve precision in determining optimal cultivation environments for different olive varieties.

**Figure 6:**
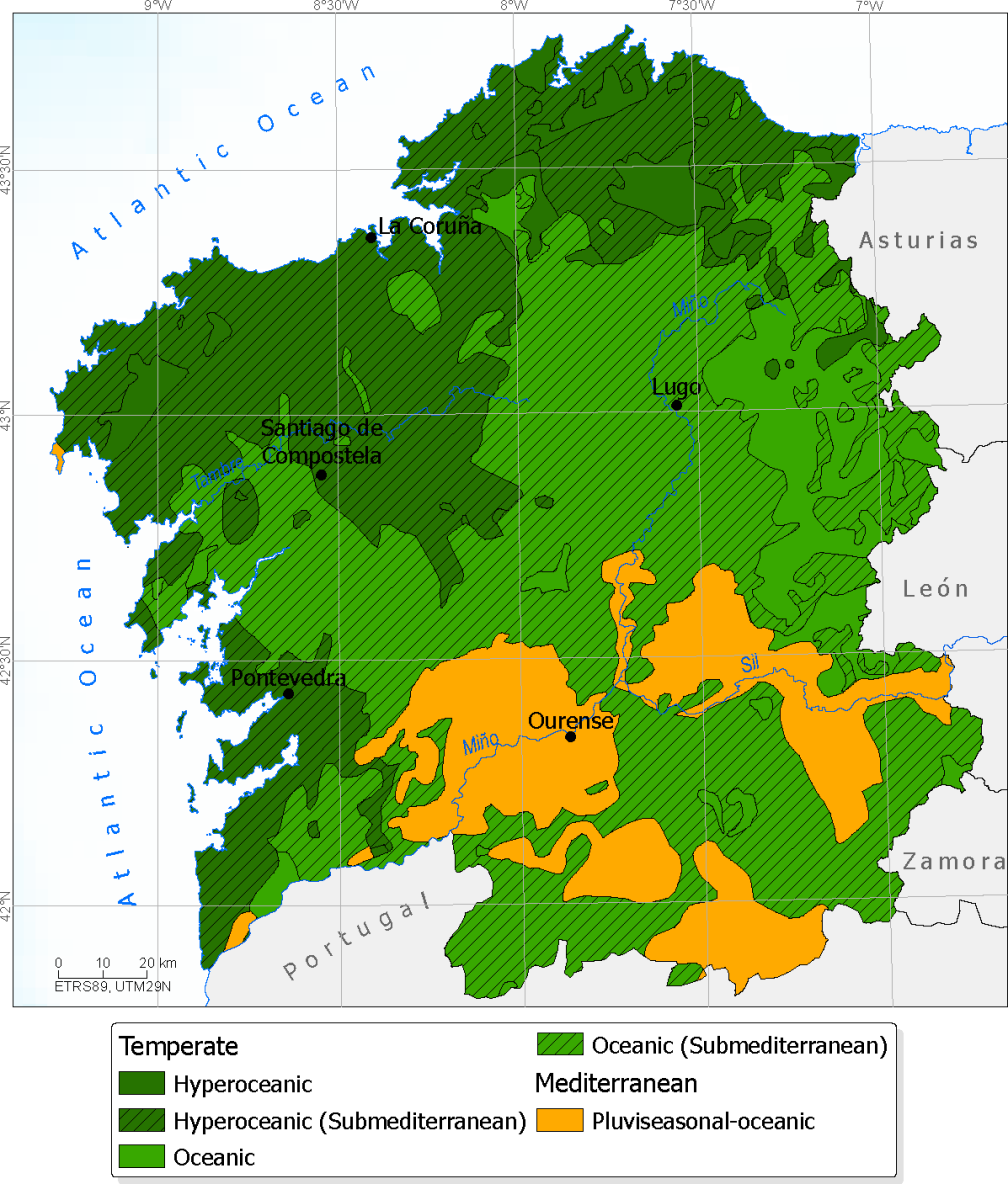
Bioclimatic map of Galicia (north-western Iberian Peninsula), showing the different bioclimates present in the region. Adapted from Rivas-Martínez et al. (2017).

Based on the hierarchical clustering and the bioclimatic characteristics of Galicia, the varieties of Group I (Brétema and Carapucho), which are better adapted to salinity and wind stress, may be particularly suitable for cultivation in coastal areas characterized by a Temperate macrobioclimate with submediterranean bioclimatic variant. These areas exhibit low continentality and high oceanicity, corresponding to a hyperoceanic Temperate bioclimate (**Figure 6**), with the thermotemperate belt being the most appropriate thermotype.

The remaining varieties (Hedreira, Cobrancosa, and Mansa Gallega) may be better suited to more continental areas, farther from the coast and at higher elevations. Bioclimatically, they could be cultivated in Mediterranean macrobioclimates (Mediterranean oceanic pluviseasonal bioclimate) or in Temperate oceanic bioclimates with a submediterranean variant.

Mediterranean macrobioclimatic areas in southern Galicia (Pontevedra, Lugo, and Ourense provinces; **Figure 6**) are particularly suitable for varieties adapted to summer drought stress. Histological traits suggest that Hedreira, with higher trichome density and longer trichome arms, may be better adapted to Mediterranean conditions, whereas Mansa Gallega, with thicker palisade chlorophyll parenchyma, may also show enhanced adaptation. Varieties with lower trichome density and thinner palisade chlorophyll parenchyma would be better suited to Temperate oceanic submediterranean areas. The most suitable thermotypes for these varieties are mesomediterranean and mesotemperate.

Considering future climate change scenarios, environmental stresses typical of xerophytic crops such as olive—high solar radiation, elevated air temperatures, high vapor pressure deficits, and limited water availability—are expected to intensify (Fischer et al., 2001; Centritto, 2002; Bacelar et al., 2004; Moriondo et al., 2013; Hmmam et al., 2018). Knowledge of the bioclimatic requirements of olive varieties and their optimal cultivation zones can therefore support the development of adaptive strategies and policies to mitigate climate change impacts on olive production.

## Conclusions

This microscopic study of fully expanded leaves from five *Olea europaea* varieties sampled in Galicia revealed significant anatomical differences among varieties. Although all varieties share the typical xeromorphic features of olive leaves, two distinct anatomical groups were identified.

Group I (Brétema and Carapucho) shows thicker leaves and cuticles, higher trichome density, thicker palisade chlorophyll parenchyma, and smaller intercellular spaces. Group II (Hedreira, Cobrancosa, and Mansa Gallega) presents thinner leaves, lower trichome density, and a more developed vascular bundle sheath.

These differences highlight the histological variability of olive leaves and provide new information for the botanical characterization of Galician olive varieties. When interpreted together with bioclimatic information, these anatomical patterns may help explore the environmental suitability of different varieties in Galicia and neighboring regions.

Future studies integrating microscopic, physiological, and agronomic approaches will be necessary to clarify the functional significance of the anatomical traits described here and their role in varietal adaptation under changing climatic conditions.

## Data Availability

The original contributions presented in the study are included in the article/supplementary material. Further inquiries can be directed to the corresponding author.
